# The epidemiology of respiratory syncytial virus: A retrospective review from Steve Biko Academic Hospital 2013 - 2016

**DOI:** 10.7196/AJTCCM.2017.v24i1.163

**Published:** 2018-04-03

**Authors:** C X Dearden, A C Jeevarathnum, J Havinga, R J Green

**Affiliations:** 1 Department of Paediatrics and Child Health, Faculty of Health Sciences, University of Pretoria and Steve Biko Academic Hospital, Pretoria, South Africa; 2 Department of Medical Virology, Faculty of Health Sciences, University of Pretoria and Steve Biko Academic Hospital, Pretoria, South Africa

**Keywords:** paediatrics, RSV, bronchiolitis, Steve Biko Academic Hosspital, South Africa

## Abstract

**Background:**

Respiratory syncytial virus (RSV) bronchiolitis is a seasonal disease that has an enormous burden on health systems across the world. RSV disease manifestations in children range from mild upper respiratory tract infections to severe lower respiratory tract infections, including pneumonia and bronchiolitis. In South Africa, the seasonality of RSV disease causing both upper and lower respiratory tract illness is well documented.

**Objectives:**

To describe the incidence of RSV bronchiolitis among patients ≤24 months of age who presented to a tertiary institution with a diagnosed viral bronchiolitis over a 4-year period. Secondary aims included determining: (i) the risk factors for the development of RSV bronchiolitis; (ii) the fatality rates and risk factors associated with mortality; (iii) the correlation with c-reactive protein values and risk of comorbid bacterial infection; and (iv) the impact of seasonality on RSV incidence.

**Methods:**

A retrospective chart-based analysis of laboratory-confirmed RSV cases in children ≤24 months, presenting to Steve Biko Academic Hospital from January 2013 to December 2016, was undertaken. Epidemiology, risk factors and local weather data were collected as part of the analysis.

**Results:**

During the 4-year period, a total of 1 127 nasopharyngeal aspirates (NPAs) was collected. RSV was isolated from 162 NPAs by either immunofluorescence (84%) or polymerase chain reaction (16%). Of the 162 patients with RSV bronchiolitis, 131 (80.9%) had a known HIV status. Only 2 (1.5%) of the patients whose status was known were HIV-infected; 26 (19.8%) were HIV-exposed and confirmed negative; and 103 (78.6%) HIV-unexposed. Forty-nine patients (30.2%) with RSV required intensive care unit (ICU, either paediatric or neonatal) admission. Thirty-four (69.4%) of these were <6 months old. Prematurity (27.8%) and cardiac lesions (13%) were the most common risk factors for acquiring the disease identified in patients with RSV bronchiolitis.

**Conclusion:**

RSV is still a commonly detected virus among infants who are admitted for bronchiolitis. Significant risk factors associated with admission due to RSV bronchiolitis were prematurity, being <6 months of age and congenital cardiac disease. Male gender and HIV status did not appear to increase the risk of RSV bronchiolitis. In fact, HIV seems to have a protective effect against specifically RSV bronchiolitis in children <2 years of age. Young babies, especially premature infants with RSV bronchiolitis, are at considerable risk of requiring ICU admission, which leads to a significant increase in admission costs.

## Background

Bronchiolitis is a clinically diagnosed lower respiratory tract viral infection characterised by wheezing and tachypnoea. The highest incidence is among children <2 years old. The pathophysiology of bronchiolitis is that of acute inflammation, oedema and necrosis of the small airway epithelial cells, increased mucus production and bronchospasm.^[1]^

Bronchiolitis can be caused by a host of viruses, all leading to a similar clinical syndrome. The viruses commonly isolated include respiratory syncytial virus (RSV), rhinovirus, influenza, parainfluenza, adenovirus, human metapneumovirus, coronavirus and bocavirus.^[2]^

High-risk children with RSV-associated lower respiratory tract infections (LRTIs) are more likely to be admitted to an intensive care unit (ICU) and have a longer hospital stay than otherwise-healthy children.^[3]^

HIV-infected children are more likely to be diagnosed with pneumonia than with bronchiolitis (p<0.01).^[4]^ In a South African (SA) study reported in 2012 that considered 105 hospitalised children <2 years of age with LRTIs, RSV was not identified in any HIV-infected cases (*n*=15) compared with 30.6% of HIV-uninfected cases (*n*=85; *p*=0.013), and was identified more frequently in bronchiolitis than in pneumonia cases (43.8% v. 12.3%; p<0.01). This might indicate that HIV infection is protective against RSV and bronchiolitis.^[4]^

Prematurity, low birth weight, being male, maternal smoking, having siblings, a history of atopy, a lack of breastfeeding and household overcrowding (>7 persons) have been observed to be significantly associated with RSV-associated acute LRTIs.^[5]^

In Pretoria, SA, the RSV season peaks in autumn (April - May).^[3]^ RSV follows a temporal trend, while other viruses are more equally distributed over the year.^[6]^ The role of the environment in the spread of respiratory infections is poorly understood. An environmental influence on RSV transmission is required to maintain this seasonality, and to dictate the timing of seasonal epidemics.^[7]^

Bronchiolitis is a viral disease. Bacterial coinfection is rare in true viral bronchiolitis.^[2]^ Blood tests are not needed routinely.^[8]^ In a study by Korppi,^[9]^ using a c-reactive protein (CRP) value of 40 mg/L as a screening limit seemed to be the most reliable method in differentiating between bacterial and viral respiratory infection. The routine use of antibiotics for mildly and moderately ill children with bronchiolitis is discouraged, because significant bacterial coinfection is rare.^[2]^

The long-term outcome of patients who have had RSV bronchiolitis is currently a topic of much debate. There is evidence that RSV bronchiolitis may predispose patients to recurrent episodes of wheezing, and possibly even asthma.^[8]^

The estimated global case fatality rate among children <1 year of age with severe RSV acute respiratory infection is 6.6 %.^[5]^

## Objective

The aim of this study was to describe the incidence of RSV bronchiolitis among patients ≤24 months of age who presented to a tertiary institution with a diagnosed viral bronchiolitis over a 4-year period. Secondary aims included determining:

- the risk factors for development of RSV bronchiolitis;
- the fatality rates and risk factors associated with mortality;
- the correlation with CRP values and risk of comorbid bacterial infection; and
- the impact of seasonality on RSV incidence.

## Methods

This was a retrospective study of all children ≤24 months old who were seen in an outpatient department, or admitted to the Steve Biko Academic Hospital (SBAH), with proven viral bronchiolitis from January 2013 to December 2016.

At the SBAH there is a well-established guideline on how to diagnose and investigate children with bronchiolitis. Every child with a clinical diagnosis of bronchiolitis has a nasopharyngeal aspirate (NPA) collected and sent for investigation. This guideline remained unchanged for the duration of the study.

Demographics (age, sex), known risk factors, HIV status, laboratory data (CRP, procalcitonin, full blood count and differential, blood culture), viral coinfection and length of stay in neonatal and paediatric ICU (PICU) and/or the paediatric wards were recorded. All positive RSV NPAs were obtained from the Department of Medical Virology at SBAH. Files were then traced through the records department to gather the study information retrospectively.

CRP and PCT cut-offs of 40 mg/L and 1 ng/mL were used, respectively.^[9]^ Either immunofluorescence or polymerase chain reaction (PCR) testing was conducted on NPA samples. HIV ELISA or PCR results were obtained from either the patients’ files or from the national laboratory website, Labtrak. Local weather data were obtained from the weather bureau to identify any possible association between RSV incidence and weather patterns (humidity, rainfall and temperature). Children from both surgical and medical wards were included in this study.

Statistical analysis was done by means of descriptive statistics utilising Stata version 15 (StataCorp, USA) software.

Ethics approval to conduct the current study was obtained from the University of Pretoria’s Ethics Committee (ref. no. 78/2017), as was consent from SBAH.

## Results

Over the 4 years studied (2013 - 2016), a total of 1 127 NPAs were conducted in children ≤24 months. A total of 288 viruses were isolated from 271 positive NPAs – in 17 NPAs, more than one virus was isolated.

The most commonly identified viruses were RSV, adenovirus, parainfluenza 1, 2, 3 and 4, human metapneumovirus, influenza, rhinovirus and bocavirus. RSV was by far the most common, isolated in 162 (14.4%) children with bronchiolitis, followed by adenovirus (4.1%) and parainfluenza 3 virus (2.1%). Table 1 depicts the distribution of viruses isolated over the 4-year period, while Table 2 demonstrates the distribution of RSV bronchiolitis over 4 years.

**Table 1:** Distribution of viruses isolated over the study period

**Virus**	**Positive results, *n***	**NPAs performed, %**
RSV	162	14.4
Adenovirus	46	4.1
Parainfluenza 3	30	2.7
HMPV	16	1.4
Influenza	13	1.2
Parainfluenza 1	7	0.6
Parainfluenza 4	5	0.4
Rhinovirus	5	0.4
Parainfluenza 2	3	0.3
Bocavirus	1	0.1
**Total**	**288**	**100**

HMPV = Human metapneumovirus

RSV = respiratory syncytial virus

**Table 2:** RSV isolates per year

**Year**	**NPAs, *n***	**RSV, %**
2013	348	51, 14.7
2014	275	42, 15.3
2015	222	20, 9.0
2016	282	49, 17.3
**Total**	**1127**	**162, 14.3**

RSV = respiratory syncytial virus

NPAs = nasopharyngeal aspirates

**Fig. 1:**
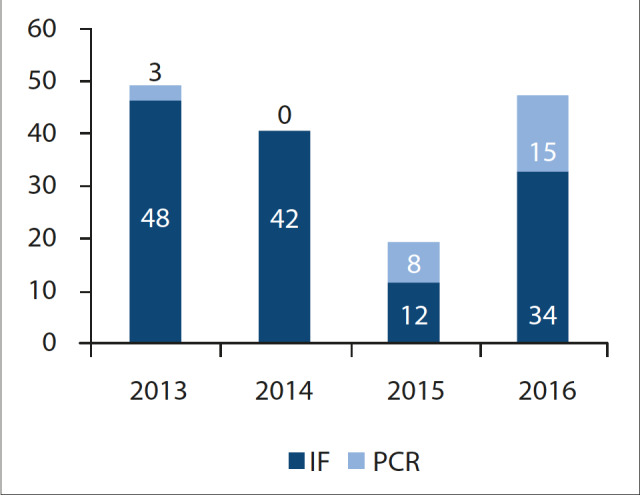
Immunofluorescence (IF) and polymerase chain reaction (PCR) use in detecting respiratory syncytial virus bronchiolitis, 2013 - 2016.

Multiplex PCR was only used frequently in the latter 2 years of the study (2015 and 2016; 3 uses of PCR were outsourced to private practice in 2013). Of the 162 RSV-confirmed bronchiolitis NPAs undertaken, 136 (84%) were conducted by immunofluorescence and 26 (16%) by PCR. As seen in Fig. 1, the total number of RSV isolates in 2015 did not increase, but rather decreased compared with previous years, despite the introduction of PCR testing.

**Fig. 2:**
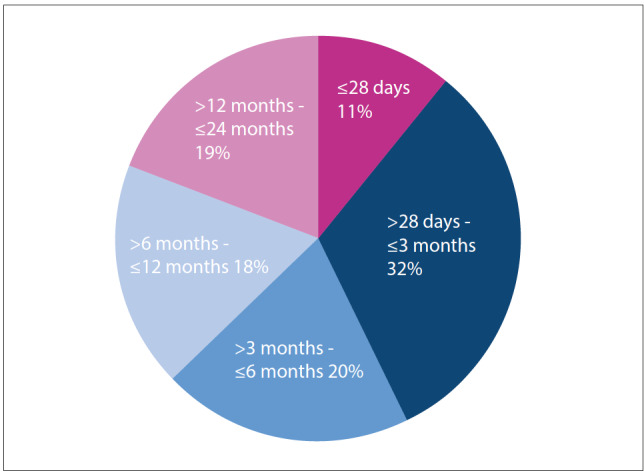
Age distribution of respiratory syncytial virus-confirmed bronchiolitis patients.

**Fig. 3:**
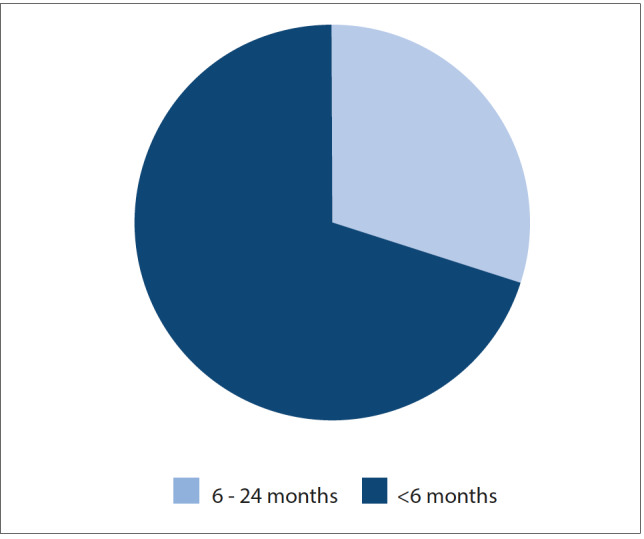
Age distribution of respiratory syncytial virus patients admitted to intensive care unit.

Of the RSV cases there were 82 male and 80 female patients, giving a male-to-female ratio of 1.03:1.00. The median age was 3.7 months (range 9 days - 2 years), with 43.8% being <3 months and 63.4% <6 months (Fig. 2). A total of 131 (80.9%) patients had a known HIV status. Only 2 (1.5%) of those whose status was known were HIV infected, 26 (19.8%) HIV exposed and confirmed negative and 103 (78.6%) HIV unexposed. Forty-nine (30.2%) of the total number of RSV-confirmed bronchiolitis patients required PICU admission. There were 34 (69.4%) <6 months old (Fig. 3).

**Fig. 4:**
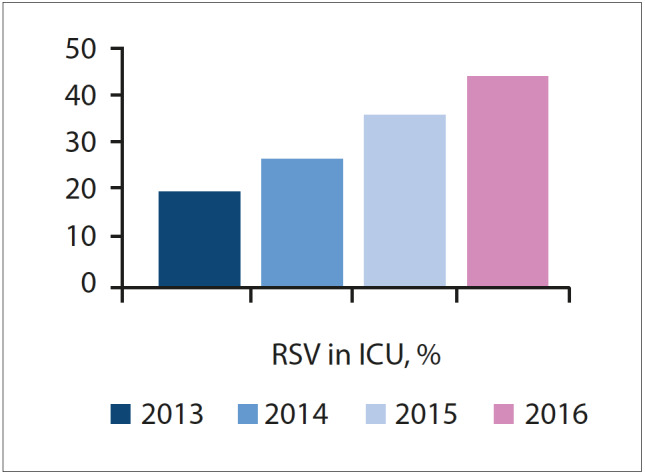
Percentage of patients with respiratory syncytial virus (RSV) admitted to intensive care unit (ICU) each year.

There was a linear increase in the percentage of patients needing PICU every year, from 19.6% in 2013 to 42.9% in 2016 (Fig. 4). There was no change in PICU bed availability during this time.

The time patients with RSV bronchiolitis spent in PICU ranged from 1 - 32 days, with an average of 9.5 days per patient. Patients who spent time in the paediatric wards stayed for an average of 6.7 days.

Of the 162 patients, there were 8 deaths (4.9%). Seven of these patients were <6 months old. Six of the patients who died were HIV-negative, while the other 2 patients had an unknown HIV status. Of the 8 deaths, 2 of the patients had both Down’s syndrome and an atrioventricular septal defect (AVSD), 1 only had an AVSD, 2 were premature and 1 patient had holoprosencephaly.

### Risk profiles

Within the group of 162 patients with RSV bronchiolitis, prematurity, followed by cardiac lesions, were the most common risk factors identified (Table 3). Chronic lung disease included bronchopulmonary dysplasia and bronchiolitis obliterance in this study. Of the 49 patients who required PICU admission, 18 (36.7%) were premature babies (Table 4). HIV did not appear to be a risk factor to contracting RSV disease as only 2 (1.5%) of the 131 patients with known status were HIV-positive.

**Table 3:** Risk factors identified in RSV-infected patients

**Risk factors**	**Patients, *n *(%)**
Premature	45 (27.8)
Cardiac disease	21 (13.0)
Chronic lung disease	12 (7.4)
Down’s syndrome	8 (4.9)
Malignancy	3 (1.9)
Mother/caregiver smoking	3 (1.9)
Sickle cell anaemia	1 (0.6)

RSV = respiratory syncytial virus

**Table 4:** Risk factors identified in patients requiring PICU admission

**Risk factor**	**Patients, *n *(%)**
Premature	18 (36.7)
Cardiac disease	7 (14.3)
Chronic lung disease	5 (10.2)
Down’s syndrome	2 (4.1)

PICU = paediatric intensive care unit.

### Laboratory data

Five RSV bronchiolitis patients had positive blood cultures for potential pathogens: Two *Escherichia coli*, one *Candida lypolytica*, one *Staphylococcus hominis* and one *Enterococcus faecalis*. Three of these five patients had a positive CRP. Three were in PICU. Two patients had additional risk factors, including gastroschisis and Hirschsprung disease. Four of the five patients were <3 months old.

A total of 64 patients had negative blood cultures, but only 61 of these also had CRPs conducted. Twelve (19.7%) of the 61 patients had a positive CRP (>40 mg/L). Of these 12 patients with positive CRPs but negative blood cultures, 7 were ventilated and had additional risk factors. Of the 64 patients with negative blood cultures, 27 had a PCT test done. Ten of the 27 PCTs were positive (≥1 ng/mL). Of these 10 patients with positive PCTs, 8 were ventilated and 6 had additional risk factors

### Weather influence

**Fig. 5:**
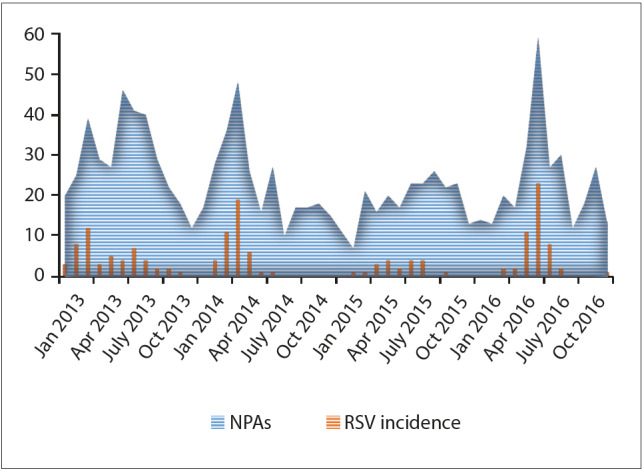
Respiratory syncytial virus (RSV) incidence compared with total nasopharyngeal aspirates (NPAs).

As shown in Fig. 5, RSV bronchiolitis at SBAH revealed an autumn and winter predominance, with an increased incidence in February - March 2013, March - April 2014, March - July 2015 and May - July 2016. Therefore the higher incidences appear to be slightly later in the year in each year of this study. In 2013, March had the most RSV NPAs isolated, while by 2016 the highest number of RSV isolates was identified in June.

**Fig. 6:**
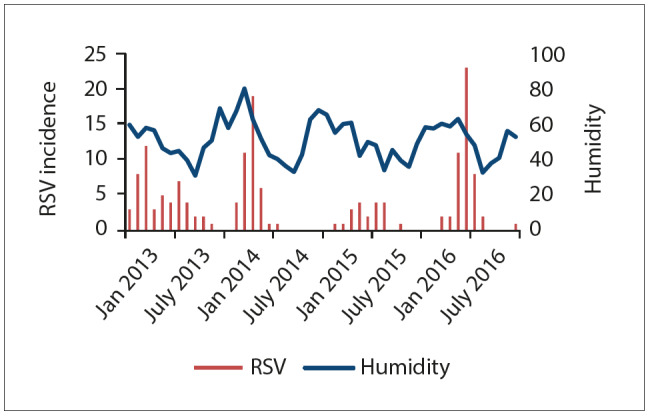
Influence of humidity on respiratory syncytial virus (RSV) incidence.

**Fig. 7:**
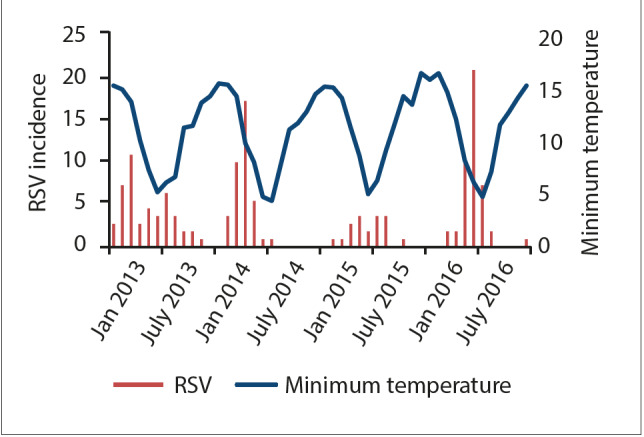
Influence of minimum temperature on respiratory syncytial virus (RSV) incidence.

**Fig. 8:**
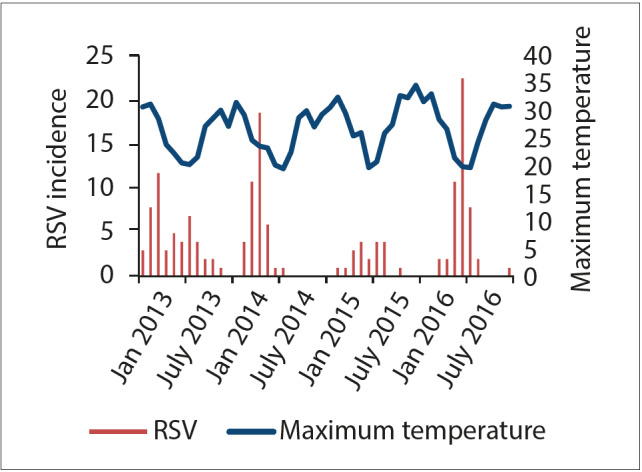
Influence of maximum temperature on respiratory syncytial virus (RSV) incidence.

**Fig. 9:**
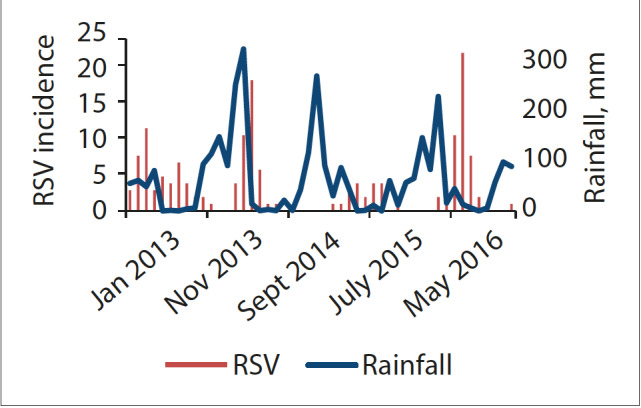
Influence of rainfall on respiratory syncytial virus (RSV) incidence. (mm = millimetres.)

Figs 6 - 9 show the effects of humidity, rainfall and temperature on RSV seasonality. Across the 4 years, there was a weak negative correlation between RSV incidence and rainfall (*r* = –0.1), minimum temperature (*r* = –0.3) and maximum temperature (*r* = –0.5), and a weak positive correlation between RSV cases and humidity (*r* = 0.3)

## Discussion

RSV was the most common virus detected during the study, and was found in 162 (14.4%) of the NPAs undertaken at SBAH during 2013 - 2016. RSV detection frequency was followed by adenovirus (4.1%) and parainfluenza 3 virus (2.1%). These results probably reflect a true viral incidence, as NPAs are carried out on all children with clinically suspected bronchiolitis.

In 2013 and 2014, virus detection was almost exclusively done by immunofluorescence. PCR testing commenced in 2015, and continued in 2016. It had been expected that detection rates would surge in 2015 and 2016, but in contrast, 2015 had markedly lower rates of RSV compared with other years, while 2016 was within the average detection rate. This suggests that the yearly changes in RSV detection cannot be attributed to the introduction of PCR testing.

There were 82 male and 80 female patients, an almost equal male to female ratio of 1.03:1.00. Forty-five of these patients were premature or ex-premature babies, of whom the male: female ratio was 0.80:1.00. This finding does not support the belief that male gender is a risk factor for bronchiolitis.

Forty-three percent of patients with RSV bronchiolitis were <3 months and 63.4% <6 months old. This corresponds with global data that suggest that infants <6 months old are at increased risk of contracting RSV bronchiolitis. Children within this age range should be targeted as part of prevention strategies.

Of the 131 patients with known HIV status, only 2 (1.5%) were HIV infected. Examining the results from a study undertaken by Annamalay *et al*. RSV was not identified in any HIV-infected children <2 years old with bronchiolitis. One could even conclude from this that HIV protects against RSV and bronchiolitis.

Thirty percent of RSV-confirmed bronchiolitis patients required PICU admission, of whom 69.4% were <6 months old. Young babies with RSV bronchiolitis are at considerable risk of requiring PICU admission, which leads to a significant increase in admission costs.

Although the admission criteria and number of beds at Steve Biko Paediatric PICU has remained unchanged, there was a linear increase in the percentage of patients requiring PICU over the course of the study, from 19.6% in 2013 up to 42.9% in 2016.

The case fatality rate of the 162 patients with RSV bronchiolitis was 4.9% (8 deaths). Seven of these patients were <6 months old. Of the 8 deaths, 6 patients had significant risk factors, re-emphasising the importance of the prevention of RSV in high-risk children.

It is well documented in many studies that prematurity, cardiac lesions and chronic lung disease are risk factors for contracting severe RSV disease. This was once again reaffirmed in this study. A total of 27% of all RSV-infected patients were premature, whilst 36% of all patients admitted to ICU were premature. This highlights the need for better prevention of RSV disease in high-risk babies by means of immunoglobulins (palivuzimab) or vaccination.

It is well documented that routine CRP and blood cultures do not contribute to the routine management of bronchiolitis, nor assist with the need for antibiotics. This study was not designed to study this effect; however, in severely ill patients that require PICU admission, it might be beneficial to use a CRP value of >40 mg/mL or PCT >1 ng/ mL to help guide the need for antibiotic treatment.

RSV bronchiolitis at SBAH revealed an autumn and winter predominance, with the peak incidence occurring later each year: in 2013, March had the most RSV NPAs isolated, and by 2016 the most RSV isolates were found in June.

Across the 4 years, there were only weak correlations between RSV incidence and rainfall, minimum temperature, maximum temperature and humidity. An environmental influence on RSV transmission is needed to maintain its seasonality, but the exact mechanisms involved (whether host, pathogen, or environment) are still poorly understood.

Some strengths of this study include the large number of patients’ files that was analysed. Good record keeping facilitated adequate interpretation of the data. The availability of weather-pattern records during the study period allowed correlations to be drawn with seasonality.

Unfortunately, the study is limited by the disparity between the lab specimens sent, i.e. NPAs and immunofluorescences. The study would have been strengthened if there was uniformity in this aspect. Rhinovirus bronchiolitis is probably under-represented because of this disparity in testing.

## Conclusion

It is beyond doubt that premature babies and infants <6 months old with RSV bronchiolitis are at increased risk for hospital and ICU admission. High risk of mortality and the severe cost implications in these patients necessitates the implementation of prevention strategies. With RSV vaccinations still in the trail phases, the only currently available prevention method is that of palivizumab, currently not available to the vast majority of high-risk patients who desperately need it. Even though palivizumab is not seen as a costeffective solution, its implementation will prevent the deaths of an uncountable number of precious infants.

RSV has a seasonal pattern, but the mechanisms involved are not completely understood.
